# Dental 3D-Printing: Transferring Art from the Laboratories to the Clinics

**DOI:** 10.3390/polym13010157

**Published:** 2021-01-04

**Authors:** Sangeeth Pillai, Akshaya Upadhyay, Parisa Khayambashi, Imran Farooq, Hisham Sabri, Maryam Tarar, Kyungjun T. Lee, Ingrid Harb, Stephanie Zhou, Yifei Wang, Simon D. Tran

**Affiliations:** 1 McGill Craniofacial Tissue Engineering and Stem Cells Laboratory, Faculty of Dentistry, McGill University, 3640 University Street, Montreal, QC H3A 0C7, Canada; sangeeth.pillai@mail.mcgill.ca (S.P.); akshaya.upadhyay@mail.mcgill.ca (A.U.); parisa.khayambashi@mail.mcgill.ca (P.K.); hisham.sabri@mail.mcgill.ca (H.S.); maryam.tarar@mail.mcgill.ca (M.T.); kungjun.lee@mail.mcgill.ca (K.T.L.); ingrid.harb@mail.mcgill.ca (I.H.); stephanie.zhou@mail.mcgill.ca (S.Z.); yifei.wang6@mail.mcgill.ca (Y.W.); 2 Faculty of Dentistry, University of Toronto, Toronto, ON M5S 1A1, Canada; drimranfarooq@gmail.com

**Keywords:** dentistry, digital diagnosis, 3D printing, additive manufacturing

## Abstract

The rise of three-dimensional (3D) printing technology has changed the face of dentistry over the past decade. 3D printing is a versatile technique that allows the fabrication of fully automated, tailor-made treatment plans, thereby delivering personalized dental devices and aids to the patients. It is highly efficient, reproducible, and provides fast and accurate results in an affordable manner. With persistent efforts among dentists for refining their practice, dental clinics are now acclimatizing from conventional treatment methods to a fully digital workflow to treat their patients. Apart from its clinical success, 3D printing techniques are now employed in developing haptic simulators, precise models for dental education, including patient awareness. In this narrative review, we discuss the evolution and current trends in 3D printing applications among various areas of dentistry. We aim to focus on the process of the digital workflow used in the clinical diagnosis of different dental conditions and how they are transferred from laboratories to clinics. A brief outlook on the most recent manufacturing methods of 3D printed objects and their current and future implications are also discussed.

## 1. Introduction

Three-Dimensional (3D) printing, also known as rapid prototyping (RP) or additive manufacturing (AM), involves the actual layer by layer addition of a material to fabricate an object or a structure using computer-aided designs and computer-aided manufacturing (CAD/CAM) technology or using advanced imaging and scanning [1]. This results in the development of custom-made products and devices for various fields, including medicine and dentistry. Although 3D printing has been used in industrial manufacturing for decades now, the technique and equipment used were fairly expensive and laborious in the past [2]. With improvements in technology, such as increased precision, high-resolution imaging, and state of the art 3D printers, 3D printing has now become a mainstream technique used across different fields today [3]. The rise of 3D printing in dentistry has been parallel with CAD advancements and enhanced imaging techniques like cone beam computed tomography (CBCT) and magnetic resonance imaging (MRI) to plan and print dental and maxillofacial prosthesis to restore and replace lost structures [4]. Previously, dentistry was mainly influenced by the process of subtractive manufacturing, also known as milling [5]. However, it did not take into account the internal structure and hence could not reproduce the complex models in its entirety [5]. Nowadays, modern CAD software is available, which uses intricate algorithmic designs and artificial intelligence to aid in modelling any object or tissue and reproducing it exactly as the clinician desires [6].

3D printing has been used for a number of clinical applications in medicine and dentistry. In the field of medicine, the use of 3D printing to synthesize customized scaffolds for bone regeneration is perhaps its most important use [7]. With the advancement in its technology, nowadays it is possible to fabricate 3D printed scaffolds for tissue engineering with tailored architecture, shape, cellular response and mechanical strength [7]. There has been an increase in the use of 3D printing for various dental applications and our review summarizes this information exactly. In dentistry, diagnosing the patient is equally, if not more important than the treatment itself. Digital dentistry has evolved as a boon in this aspect, which assists the dental practitioners in quickly and precisely recording and evaluating the dentofacial structures [8]. This allows us to bypass several manual techniques and replace them by using automated devices such as intra-oral scanners and advanced imaging, thus minimizing the chance of errors. Today, most dentistry disciplines utilize digital scanning, designing and layered manufacturing techniques to provide patient-specific custom-made prosthesis and restorations [9]. This review discusses how this digital workflow materializes into the fabrication of diverse dental and orofacial prosthesis across various disciplines in dentistry. We have searched PubMed, OVID and MEDLINE databases for clinical studies and cases reporting the use of 3D printing technology in dentistry using combination of multiple keywords that included ‘dentistry’, or ‘Endodontics’ or ‘Prosthodontics’ or ‘Orthodontics’ or ‘Endodontics’ or ‘Oral surgery’ or ‘Periodontics’ or ‘dental implantology’ and combined with ‘3D printing’ or ‘CAD/CAM’ or “Additional manufacturing” and combined with ‘digital dentistry’ or ‘diagnosis’ or ‘scanning’. We identified the techniques used, current trends in treatment protocol, the challenges faced, and their future within dentistry.

## 2. Digital Workflow from Clinical Diagnosis to Treatment Delivery

**Figure 1:**
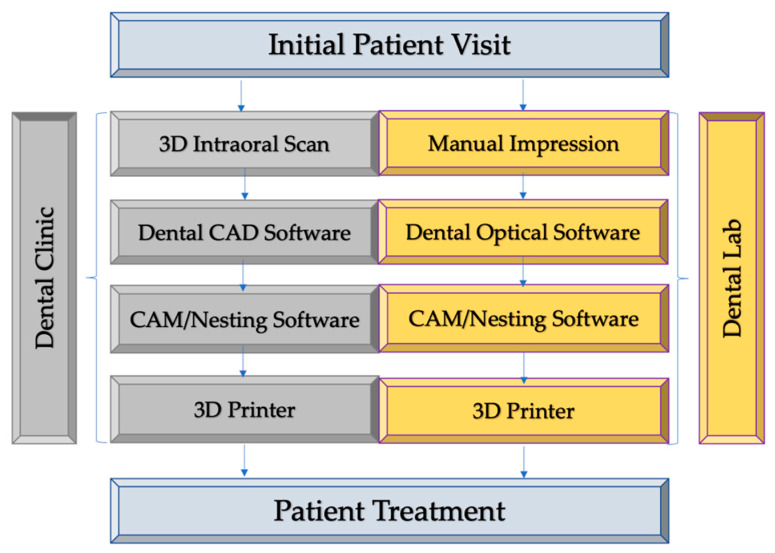
Digital workflow of dental diagnosis and treatment using CAD/CAM and 3D printing technology.

Digital dentistry is an umbrella term for various digital technologies such as the use of precise intraoral scanners, 3D imaging aids, CAD/CAM software and 3D printers that can enhance and increase efficiency as compared to traditional analog techniques such as impression making, 2D imaging, and conventional subtractive manufacturing techniques. These technologies can be incorporated into many procedural workflow stages and can be used in combination with traditional methods. Virtual planning is increasingly being used in tandem with 3D printing during the pre-treatment/pre-surgical phase to improve patients’ treatment outcomes [10]. The virtual planning process starts with an accurate capture of a patient’s anatomy by using 3D intraoral scanners (Figure 1). While different intraoral scanners may vary in performance, clinically acceptable accuracy has been shown to be consistently achievable [11]. Alternatively, traditional impressions/plaster models can be sent to dental labs and scanned with desktop optical scanners. The patient data obtained by scanners can then be transferred to CAD software for treatment planning and digital design of 3D models. Today’s dental software can employ highly visual interfaces and familiar design processes for virtual setups, such as for smile design [12].

After the treatment design, 3D models can be exported for manufacturing with ease using CAM software and subsequently sent to a 3D printer. By working in tandem with the precision of digital 3D models, 3D printers can be employed by both clinical practices and labs to create a variety of products for treatment, such as dentures, surgical guides and splints, aligners, retainers, and mock-ups [13].

## 3. 3D Printing Techniques Used in Dentistry

Currently, the availability of several printing techniques and machines allows a wide range of applications of 3D printing both in the industry and medicine. However, in dentistry, the most commonly used 3D printing methods include stereolithography (SLA), fused deposition modelling (FDM), selective laser sintering (SLS) and digital light processing (DLP) [14].

**Figure 2:**
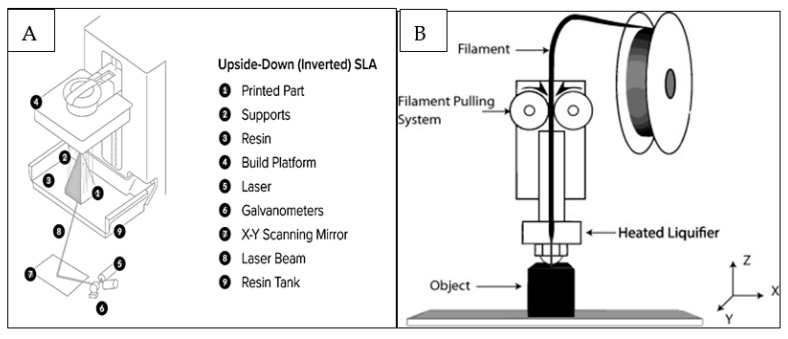
(**A**) Prototype representations of Stereolithography (SLA/SLG) 3D printing method, adapted from the *dental.formlabs* website [18] and (**B**) Fused deposition modelling (FDM) printing technique. Image reprinted with permission from Carneiro et al. [17].

- Stereolithography (SLA/SLG) (Figure 2A) is a 3D printing process that uses mirrors and, by motorizing them, selectively moves an ultraviolet (UV) light beam to fuse surfaces that contain photoreactive liquid resin [15]. The process is followed by a wiper recoating the cured surface and another fusion step with the possibility of staining or infiltrating the specific areas [15]. The advantages and limitations of different printing techniques are displayed in Table 1.
- Extrusion-based methods rely on a continuous (no droplets) deposit of material driven out of the nozzle by either pneumatic or mechanical forces creating 3D constructs at the centimeter scale [16]. FDM, (Figure 2B) is an extrusion-based printing technique in which thermoplastic materials are subjected to melting to develop filaments which are deposited to fabricate the desired objects [17]. They are used for quick, low cost printing of basic less intricate models which are typically machined.

- Selective laser sintering (SLS) uses a high-power pulsed laser to fuse thermoplastic polymer (or metal, ceramic and glass) particles. It creates surface layers that will be refreshed using a roller or blade. A roller or blade then refreshes each sintered surface layer using powder material. One benefit of the 3D printed models using SLS is that they are thermoplastic in nature and can be autoclaved therefore can be handled safely during dental treatments [15].
- Digital light processing (DLP) uses similar concept as SLG to create 3D prints; however, instead of a set of moving mirrors, a digital micromirror device creates the cross-sectional UV image [15]. In dentistry, use of photocurable resins are highly suited an DLP utilizes this aspect for fabrication of single layer 3D objects using UV or white light. The final print properties can be modified as desired by simply manipulating the resin characteristics [19].

## 4. Biomaterials used in Dental 3D Printing

Various 3D printing methods exist in the field of dentistry, as described in the previous section. However, along with these methods, the fabrication of 3D dental materials has been explored heavily in the literature. A wide range of biomaterials exist for 3D printing, such as hydrogels, metals, ceramics, resins, and thermoplastics. The compatibility of dental materials and their reproduction accuracy based on the 3D printing technique is described in Table 2 [24,25]. 

### 4.1. Hydrogels 

Hydrogels have been viewed as one of the ideal materials for 3D printing. Hydrogels are porous, crosslinked polymers with hydrophilic characteristics, causing them to retain water [28,29,30]. This represents an advantage as it resembles the characteristics of the native extracellular matrix (ECM). Also, hydrogels have high tunability in their biological, chemical, mechanical and rheological properties, demonstrating an elastic characteristic [31]. The printability of polymer hydrogels is defined by its viscosity. They need to be fluid enough to eject from nozzles and be viscous enough to form and support structural layers. In addition, the biological and mechanical properties of polymer hydrogels can be further advanced when combined with other approaches, including ionic interactions, light exposure and pH stimulation, further broadening their applications [31]. For example, to balance polymer hydrogels’ viscosity at an optimal state, alginate hydrogels are cross-linked with calcium ions before being ejected from the nozzle. Several combinations of hydrogels are available today such as photocrosslinkable gels, which can be generated or degraded by the exposure to ultraviolet (UV) light. Reinforced-composite or polymer-composite hydrogels, which incorporate natural or synthetic polymers or secondary polymeric networks such as, interpenetrating polymer networks (IPNs) are other examples of combined hydrogels. Other types include nanocomposite-based hydrogels which incorporates mineral, polymeric or metallic nanoparticles containing specific physical properties to the hydrogels to further reinforce the structural state of hydrogel systems [25,29,32]. In addition to naturally derived hydrogels, synthetic hydrogels, including polyacrylamide (PAM), poly (ethylene glycol) (PEG), poly (2-hydroxyethyl methacrylate) (PHEMA) and poly (vinyl alcohol) (PVA), are used in 3D printing due to their controllable properties in degradation and high mechanical characteristics.

### 4.2. Polymers and Thermoplastic Materials

Polymer-based 3D printing accounts for the most commonly used material among the variety of options available for additive manufacturing. Most 3D printers available at the dentist’s disposal today accommodate a wide range of polymeric substances which are used in the fabrication of dental implants, crowns and bridges and other 3D tissue structures [33]. Among the techniques used for utilizing resin or polymeric materials in dental 3D printing, photopolymerization is highly plausible [33]. Since dentistry is already a field utilizing photopolymerization process when manipulating patient compatible materials due to its ease and time efficiency, it provides similar advantages in 3D printed models such as better build resolution, smoother surfaces, good chemical bonds and mechanical strength [33]. Thermoplastic polymeric materials are extensively utilized in the field of 3D printing. These polymers are made from filaments that are heated as they are deposited through the nozzle, allowing the materials to be tunable for specific structures [26,34]. A variety of these materials including, polylactic acid (PLA), acrylonitrile butadiene styrene (ABS), polypropylene (PP) or polyethylene (PE) are considered suitable for oral cavity. PLA has been viewed as a more favorable material than ABS due to its high resistance against impact and non-toxic properties against oral cavity [26,35]. More recently, thermoplastic filament materials with higher melting temperatures like PEEK (polyether ether ketone) and PMMA (polymethylmethacrylate) have been used in dental 3D printing [36]. Overall, an improvement in both polymeric material properties regardless of their form as resin, powder form or filament complements with the advances in device technology in fabricating 3D printed objects in dentistry.

### 4.3. Ceramics

Ceramics represent another common material for 3D printing approaches, especially in the field of prosthetic dentistry. Ceramics are often utilized in SLA and SLS, in which specific ceramic powder or pre-sintered ceramics are targeted to create strong bonding [25,26,37,38]. Studies have also shown that incorporating calcium and phosphate mineral phases like hydroxyapatite and β-tricalcium phosphate provides ceramics an ability to form a biocompatible microenvironment. This can further allow the ceramics to develop cell-to-cell interactions and promote cell differentiation and proliferation, making them favorable for craniofacial applications [39,40]. However, due to challenges present with post-processing to high density, ceramic powder can only develop porous structures through SLS. In addition, additive manufacturing techniques themselves create limitations as sintering ceramics can lead to anisotropic shrinkage and fabricating leads to stair-step effects on surfaces. Thus, 3D printing for ceramic restorations has been limited, only being viewed in research [26,28,41].

### 4.4. Metals

Metal is another common material used in dentistry. Its popularity has been further viewed in the field of 3D printing as well, mainly in the use of SLS. In dentistry, metallic materials that were considered included titanium, cobalt-chromium (CoCr) and nickel alloys. However, researchers no longer consider nickel alloy, specifically nickel-chrome (NiCr), as a suitable material for dental prostheses due to possible nickel allergic reactions in the oral cavity. Like ceramics, fabricating metallic dental prostheses using SLS resulted in porous structures and led to using varying diameters and laser strengths. Recent research has led to further improvements of SLS techniques, such as including a vacuum during fabrication processes of metallic dental prostheses [26,42,43,44]. Titanium and CoCr are highly favourable metallic materials for 3D-printed dental prostheses. Due to their unique physical properties, including favourable levels of strength and ductility, titanium alloys, specifically Ti_6_Al_4_V, have demonstrated their capability as maxillofacial prostheses in various clinical trials [26,37,45]. Yet, research on their use has been limited due to the cost of titanium alloys, turning the focus to CoCr alloy, which present numerous advantages. Compared to other metallic materials, CoCr alloy presents lower density, higher hardness and good corrosion resistance and bonding characteristics to porcelain [26,46,47,48,49]. A study by Barazanchi et al. (2020) demonstrates that CoCr materials fabricated by SLS have a higher bonding capacity with porcelain compared to CoCr materials fabricated by soft milling. These properties further indicate the alloy’s good stability in the oral cavity and tolerance against loads, representing it as a preferred material for 3D-printed dental prostheses in long term applications [50].

Furthermore, the use of Direct Metal Laser Sintering (DMLS) on CoCr alloy to produce dental prostheses has demonstrated the elimination of issues present during milling on CoCr alloy, including the shrinking of CoCr materials during casting. This further concludes that CoCr alloy fabricated from 3D printing techniques demonstrates higher biocompatibility in the oral cavity than other metallic materials, such as NiCr alloy, used as alternatives to gold alloy in dental prostheses. Moreover, in vitro studies have demonstrated that CoCr materials fabricated by SLM still had clinically acceptable marginal gap between ceramic and metal frameworks and metal-ceramic bond strength even after ceramic firing. This further highlights CoCr alloy as a promising material for 3D printed dental prostheses using 3D printing techniques, such as SLM [26,47,49,51,52].

There are various 3D printing methods and materials that can be used commonly in clinical settings. Ceramics, such as zirconia, and metal alloy, such as CoCr, represent ideal materials to form 3D dental prostheses through SLA or SLM. Further research should be conducted to understand better the use and effectiveness of these 3D printing methods and materials in clinical settings.

## 5. 3D Printing in Endodontics

Since the late 80s, the diverse applications of 3D printing techniques have revolutionized the scope of design possibilities for creating new restorations, dental models and surgical guides, increasing the success rate of conventional surgeries dramatically [53]. Although use of 3D printing in endodontic treatments are yet to be explored, there are several reports and pre-clinical studies that describe the improvements brought in guided access, maneuvering obliterated pulp canals, auto transplantation, but most importantly in endodontic and general dental education [54].

Using a CAD/CAM-guided surgical template in endodontic surgery allows surgeons to target the root apex, especially in teeth with problematic anatomies [55]. Guided osteotomy and apex localization had been achieved using these templates in cases such as a mandibular molar with a thick buccal bone template [53]. In CBCT, the use of CAD/CAM has leveraged the data regarding the objects used in surgical or nonsurgical endodontics [54]. The anatomically challenging cases have also been defined using targeted endodontic microsurgery (EMS) using 3D printed guides, and trephine burs [55]. When estimating control of depth, diameter and angle of root-resection, targeted EMS mainly benefits osteotomy more than traditional approaches [55]. Irreversible pulpitis, pulp necrosis or apical periodontitis can now be dealt with using nonsurgical root canal treatment and EMS, which consequently relinquishes better outcomes (about 35% higher success rate) compared to the traditional techniques allowing for superior visualization, magnification and illumination [55].

Fabrication of intricate 3D features to mimic a functional extracellular matrix is another application of 3D printing in recreating craniofacial and dental tissues [55]. Even though the demand for regenerative construction is still present, the biomanufacturing methods are still limited [55]. When it comes to root canal treatments, the pulp evoked bleeding combined with the body’s clots determines the remodeling after a procedure; however, biofabrication-inspired methods such as novel bioinks had demonstrated promising results [56]. Use of decellularized extracellular matrix (dECM) as novel bioinks have been suggested as an ideal matrix structure by several authors owing to their natural composition. For example, Athirasala et al. described the synthesis of a novel bioink, Alg-Dent, using printable alginate hydrogels which are blended with different concentrations of dentin matrix. They found that high levels of alginate and insoluble dentin matrix was most suitable for cell viability and increasing the concentrations of soluble dentin matrix molecules will further improve the odontogenic differentiation potential and thus can act as ideal biomaterial in regenerative dentistry [56]. Among the techniques attentive on root canal filling approaches, filling simulated C-shaped canals have been entertained using 3D printed resins [57]. The simulated root canal model has been used to get passive and active sodium hypochlorite to remove *Enterococcus faecalis* biofilms and compare agitation levels [58]. Although use of advanced 3D printing technology is in its budding state in endodontic surgeries, careful utilization of this tool can improve the outcome of EMS and root canal treatments in patients.

## 6. 3D Printing in Prosthodontics

### 6.1. Crowns and Bridges

Restorations using crowns and bridges are among the common clinical procedures in prosthodontics. Traditionally, they were fabricated using the lost-wax technique, which is labour intensive and prone to human errors. Understandably, comparative studies for evaluating various dental restorations parameters like crowns and bridges have been performed in light of the current popularity of 3D printing technologies to predict their reliability. Mai et al. (2017) reported that milling and additive manufacturing showed more accurate results regarding marginal fit compared with manual techniques. Moreover, 3D printed crowns had the most accurate occlusal fit and least internal discrepancies [59]. Lower marginal gaps were reported by Alharbi et al. (2018) amongst all finish line designs when the models were 3D printed [60]. Similar results were obtained in other studies which are evidences that conclude that 3D printed crowns had significant marginal fit [61]. In a systematic review, the performance of CAD/CAM systems was influenced by the restorative material. Thus, the outcomes varied with inconclusive evidence of 3D printing technologies’ superiority over casting techniques and milling [62]. Prechtel et al. (2020) evaluated the mechanical properties of restorations fabricated using different techniques [63]. The study compared the fracture load of 3D printed indirect PEEK inlays with conventional milling, direct composite restoration and chewing stimulation of sound human molars with thermal cycling. Results showed that both 3D printed, and direct restorations showed superior fracture load when compared to physiological chewing forces, but sound molars had the highest load resistance. However, they also concluded that all 3D printed inlays remained intact after fracture load test and showed a more significant success as compared to conventional direct restorations [63]. Several dental materials with varying mechanical properties have been used in fabricating dental crowns. Amongst them, ceramic materials, including alumina (AlO_3_) and zirconia (ZrO_2_) ceramics, have gained popularity in 3D printing due to their high bond strength, providing unique mechanical properties to crowns and bridges. A previous study has reported that when DLP technique was used to print zirconia implants, they demonstrated sufficient dimensional accuracy [64]. Moreover, an earlier in vitro study revealed that 3D printed zirconia crowns demonstrated similar trueness of the CAD/CAM crowns. This demonstrates that zirconia is a useful material for 3D printing in the field of prosthodontic dentistry [65]. Nevertheless, the final verdict of their clinical success versus the cost involved still requires more systematic and comparative studies [63]. 

### 6.2. Fabrication of 3D Printed Dentures

The exponential growth of using digital technologies in the field of prosthetic dentistry can be mainly attributed to their application in the fabrication of removable prosthesis, such as complete and partial dentures [66]. In recent years, dentures fabricated by digital techniques have become increasingly popular. Because there is a variety of different CAD/CAM programs and protocols, the procedures for the digital manufacturing of dentures can vary, with some requiring only two appointments with the dentist [67]. In addition to the reduced chair time for patients, digital dentistry allows for the storage of electronic data, enabling technicians to precisely duplicate a denture in a matter of hours. Furthermore, variability in quality can be minimized [68]. A disadvantage of a fully digital approach is the lack of a wax try-in. This is an essential step in evaluating a denture before it is finalized. However, some approaches developed to work around this limitation, such as try-in dentures can be 3D printed or milled using low-cost materials for the patient to try on and make modifications if necessary [69]. Another approach is a virtual try-in, whereby a face scan is combined with an intraoral scan and the digital tooth set up [70].

Digitized impressions serve as the first step in a digital workflow. This can be done directly via intraoral scanning or indirectly by extraoral scanning of impressions or models made from impressions [71]. The conventional impression method involves applying alginate impression materials, which may cause mucosal irritation, gagging, and discomfort for the patient. In comparison, digital impressions have been shown to be more comfortable and require a shorter amount of time to complete and have fewer errors [72]. The digitized scan creates a digital file, such as a standard tessellation language (STL) file, that can be quickly sent to CAD software to design the denture [73]. Despite the benefits of intraoral scanning, a few disadvantages limit its application. For example, difficulties in obtaining a clear scan could arise due to saliva and blood inside the mouth [74]. Moreover, some intraoral scanning devices are not suitable for capturing soft-tissue morphology or recording the extension of mobile tissues [72]. One study analyzed the capability of intraoral scanners to reproduce accurate edentulous arches. The results showed that the digital impressions appear to be feasible, but the scanners’ accuracy differs significantly [75]. As new scanners are regularly developed and brought to market, digital scanning is expected to continue improving reliability and accuracy and eventually replace conventional impressions. The digitalization of the conventional impression is followed by CAD design of the denture using one of the plethora of CAD software programs available. The CAD software then helps with the positioning of teeth and modelling the denture base with high-level accuracy and detail. Teeth can be chosen from a vast digital library of existing denture teeth to suit the patient [69]. Once the design is complete, the denture can be fabricated.

Until recently, subtractive manufacturing was the primary method for the commercial production of dentures. However, a disadvantage of the milling process is the material wasted as a result of removing material from a block to fabricate the denture [76]. In contrast, 3D printing technology is an additive process using powder substrates that involves adding layer atop layer of light-sensitive material to produce the denture. The residual powder can be recycled for future fabrications, promising a more sustainable additive approach by reducing waste [68,76]. Usually, CAD/CAM fabricated dentures require milling or printing of the denture base first, followed by adhesion of the printed denture teeth with a bonding agent [77]. However, one in vitro study found that bonding denture teeth to conventional heat-cured denture bases produced significantly higher fracture toughness, and bond strength than teeth bonded to milled and 3D printed denture bases [78]. However, with recent developments, it is now possible to fabricate both the denture teeth and the denture base together in a single step [69]. Several studies have been conducted to evaluate dentures fabricated by CAD/CAM. In one study, patients reported that CAD/CAM dentures were more comfortable, more aesthetic, better fitted, and allowed for improved eating and speaking [79]. When asked to compare digitally made dentures and conventional compression-molded dentures, patients consistently gave higher scores for the digitally produced dentures [80]. Some authors found that digitally fabricated dentures offered better accuracy, reproducibility, and retention [81]. When comparing denture bases fabricated by milling or traditional compression molding, the denture bases produced by the digital technique were once again found to have a better fit [66].

Nowadays, improvements in oral health maintenance have resulted in fewer missing teeth, leading to a greater need to treat partially edentulous patients compared to completely edentulous [82]. The removable partial denture (RPD) is commonly used in clinical applications to restore lost teeth [83]. In some situations, a well-designed RPD is the only choice, such as in cases of long edentulous spans, lost or severely resorbed residual ridges, or absence of posterior abutments [84]. Despite advances in dental materials and technology, conventional cast partial dentures are still fabricated using the lost-wax technique. The conventional lost-wax technique is limited by dependency on a technician, time required, procedural errors, and multiple adjustments [85]. Conventional RPD frameworks are often fabricated from CoCr, which has many drawbacks, such as allergic reactions and biofilm plaque formation [86]. The relatively large casting shrinkage of the alloy also requires the expansion of the investment materials to compensate [87]. The development of CAD/CAM technologies has facilitated the production of metallic RPD frameworks [83,88]. A clinical report from 2017 reported the successful use of 3D printing to fabricate a pure titanium metal framework for an RPD of the maxillary arch [83]. A case report by Wu et al. (2020) demonstrated the use of more than one 3D printing technology to fabricate an RPD with a distal extension. Stereolithography 3D printing, which uses a UV-sensitive liquid resin as a substrate, was employed to print custom trays for a patient. In the same study, SLM was used to fabricate the metallic framework [89]. In vitro studies have found a similar fit accuracy of SLM manufactured frameworks compared to those made by the conventional lost-wax technique [84,86]. Even when compared to milling, SLM manufactured frameworks have been shown to provide a better fit [76]. 

While there are advantages and disadvantages to traditional and digital methods of fabricating dentures, the rapid advances taking place in the field are paving the path for digital dentures to become the standard. However, many clinicians and technicians still fabricate prostheses using analog methods because of the high cost of digital equipment and the time required to train staff to use it [83]. During this transitional phase between analog and digital techniques, incorporating both will be useful moving forward.

## 7. 3D Printing in Oral and Maxillofacial Surgery

The craniofacial area is considered an esthetic zone and can be affected by trauma, tumors, congenital abnormalities, facial deformities and many other diseases and oddities. Surgical reconstruction is the gold standard approach used in these situations. However, the maxillofacial region is anatomically and functionally complex and is encompassed with hard and soft tissue innervated profusely with nerves and blood vessels. Thus, the reconstruction of any defect or deformity in these regions becomes highly challenging and perplexing for the surgeon.

The initial use of 3D printing in OMFS dates back to a few decades [90]. However, their use in clinical applications boomed over the past decade owing to the evolution in technology and accessibility to low-cost 3D printers [90]. Conventional methods chiefly used autologous grafts from other parts of the body and used techniques like fibula free flap (FFF) and iliac osteocutaneous flaps to correct the defects surgically [91,92]. These techniques were effective and have shown good success in reconstructing mandibular defects [93]. However, in reconstructive surgery, bone remodeling is of utmost importance and requires precise techniques and planning to maintain the jaws and facial structures’ correct shape and anatomy. To some extent, traditional techniques can be indefinite regarding the angles, shapes or osteotomy sites and thus increase the chances of error [94]. Moreover, studies have shown a high percentage of donor site morbidity in autologous graft extraction sites leading to several complications, including graft loss, wound dehiscence, cellulitis and abscesses [95]. 

**Figure 3:**
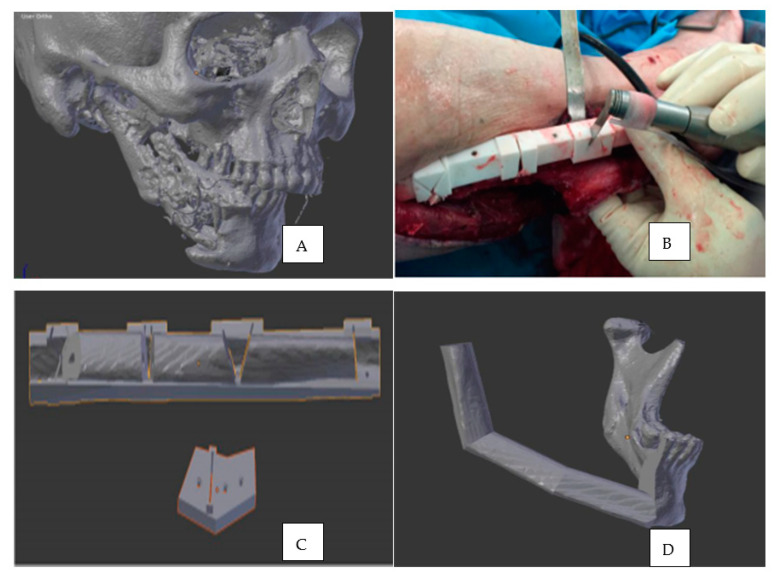
(**A**) CT of skull with osteoradionecrosis (**B**) 3D printed surgical guides used to guide osteotomies to obtain fibula from patient’s leg. (**C**) Surgical guide with harvested bone. (**D**) Surgical reconstruction of the jaws using scanning and 3D modelling. Image reprinted with permission from Ganry L et al. [99].

Today, many oral surgeons are using virtual planning and 3D printing technology to provide better care and treatment outcomes to their patients. According to a systematic analysis by Jacobs et al. (2017), four categories use 3D printing for craniomaxillofacial surgery in patients, which includes contour models (Type I), surgical guides (Type II), splints (Type III) and implants (Type IV) [96]. Developing contour models is the most common and is called a positive space model as it involves direct printing of the object based on patients’ external anatomy based on imaging [96]. These models can be developed using in-house printers and are hence more economical and time-saving in emergencies like fractures [97]. 3D printing assists in developing precise surgical guides and reconstruction plates in cases where autologous bone grafts are the primary choice of treatment to replace lost structure (Figure 3) [98]. These guides are designed and manufactured using CAD/CAM technology and are 3D printed. They act as precise tools in harvesting hard and soft tissues from donor sites to be transplanted to the deformity [99]. Type III are splints used in orthognathic correction, such as alignment of the jaws and occlusion. This is a more virtual negative space model; that is, they are constructed by virtually planning the future positions and orientation of the bone and teeth, requiring advanced 3D modelling to obtain right end results [96,97]. Implants are within the type IV category and are less commonly developed as 3D printed objects due to grander demands in the fabrication process. They are highly specific in their structural, functional and biological aspects. They are used in cranial and condylar repairs and in jaw reconstruction to provide adequate support and form.

The extent of utilizing 3D printing in oral and maxillofacial surgery is mainly determined by their application in different surgical procedures as classified earlier. In a systematic review by Louvrier et al. (2017), they graphically represented the critical areas in maxillofacial surgery, which utilized 3D printing technology the most. It showed that most surgeons used the technology to manufacture surgical guides for reconstructive surgery and implant placement [90], followed by use in mid-face and mandibular reconstruction, with orthognathic and cranial surgery being the next common procedures utilizing 3D printing technology [90]. After evaluating the recent literature, we came to a similar conclusion regarding the trend of authors using 3D printing in the most common areas in OMFS. Table 3 depicts the different studies using 3D printing based on clinical applications.

Most OMF surgeons in these clinical studies emphasize the improved precision of the procedure and better time efficiency as the key advantages of using virtual planning and 3D printing in maxillofacial surgeries. Many report the improvement in esthetics after reconstructive surgery and better post-operative facial symmetry [115]. Also, surgical guides and the development of presurgical templates provide lesser comorbidity to the donor sites. However, although the CAD/CAM technology saves valuable operating time, preoperative virtual planning takes up more time. The surgeon’s ability to use and understand the modern software and computer programs is always put in the line of question when planning surgery. This said, 3D printing is an exceedingly useful tool in dentofacial surgery. When combined with a surgeons’ skill, it can create extraordinary results in the human body’s most esthetic zone.

## 8. 3D Printing in Periodontal Surgery

Numerous attempts, often successful, have been made in the regeneration and healing of periodontal defects using guided tissue regeneration. However, many areas remain challenging like non-contained defects, namely, walled intrabony defects and horizontal bone resorption. Lack of support and site protection for grafted materials limit the treatment options and bring down the clinical success rate. Computer-aided rapid prototyping (CAPR) allows better visualization of the surgical site and aids in treatment planning. CAD/CAM is increasingly being used to generate patient models, enabling simulation of idiosyncratic contours of the tissues and defects, including clinically unapproachable areas. Computer-assisted designs offer more accurate models over which transplantation of grafts can be planned. This methodology was used by Lei et al. (2019), for contouring the graft consisting of Platelet-Derived Growth factor (PRF) over a 3D printed model of the patient [116]. They used Advanced-PRF (A-PRF), which is known for its abundance of osteoinductive and antibacterial factors, as well as Injectable PRF (I-PRF), which accelerates the solidification of A-PRF, thus providing a plastic structure conforming to the defect [116]. The use of computer-assisted devices to serve as surgical guides has been immensely used in the medical field. Yin et al. (2017) prepared such a surgical guide to rebuild the marginal contours in the anterior esthetic zone. They pre-surgically designed 3D gingival curves by using reverse engineering software. Further, they designed the crowns using the Tarnow principle [117] to induce the bone and gingiva’s structural reformation. Given the importance of gingival biological width, which, if encroached, leads to bone resorption, the proper planning leads to highly esthetic and successful outcome [118].

**Figure 4:**
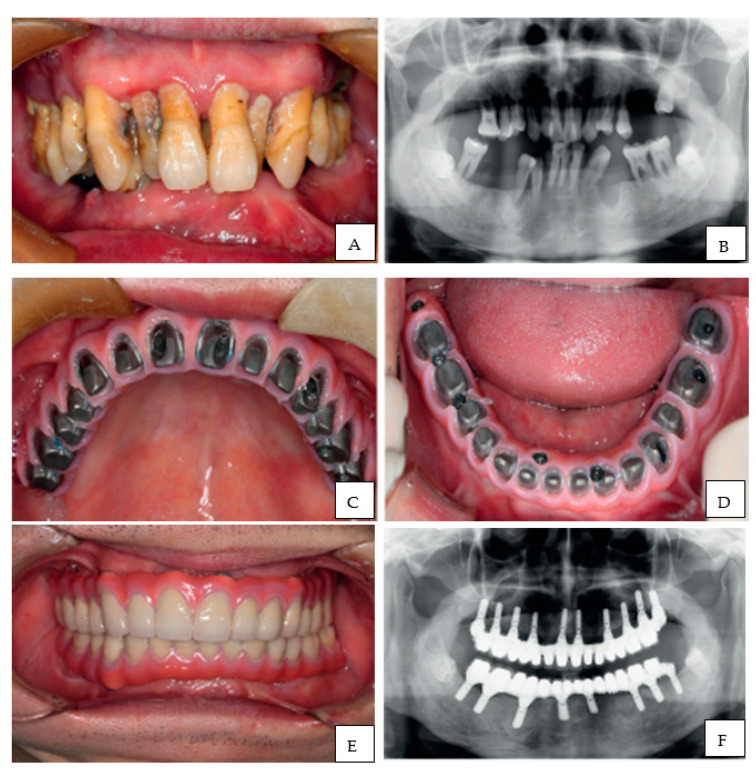
(**A**) Pre-operative condition- Intra-oral, (**B**) Panoramic radiograph, (**C**,**D**) Screw retained framework, maxillary and mandibular respectively, (**E**) Intraoral view of definitive prosthesis in place, (**F**) Panoramic radiograph after treatment. Images re-printed with permission from Wang et al. [119].

An impressive example of full mouth restoration using CAD/CAM technology in a patient with severe periodontitis and alveolar ridge atrophy is described by Wang et al. (2018) (Figure 4). They restored the atrophic arches by fabrication of 3D printed customized titanium framework over which implants were supported, leading to restoration of function as well as esthetics [119].

Autotransplantation of a tooth involves replacing a decayed/lost tooth using the patient’s own tooth with less developed roots and intact crown, usually the third molar. The underdeveloped roots ensure the development of periodontal structures naturally and aid in its integration. Quite often, the root morphologies of the replaced and the replacement tooth differ, due to which the socket shapes are different as well. Computer-assisted technologies have enabled clinicians to view, plan and prepare the surgical site to simulate and align to the intricacy of donor tooth, thus making it possible to have a less traumatic and better-adjusted tooth vs. bone interaction [120,121]. Rasperini et al. (2015), pioneered in clinical use and development of a 3D scaffold for periodontal regeneration. Inspired by in vivo studies that used polycaprolactone (PCL) scaffold for periodontal ligament (PDL) formation, they developed the scaffold by integrating SLS technology. The 3D design was created digitally to fit the defect best, further modified to contain perforations for fixation, an internal port for delivery of recombinant human platelet-derived growth factor (rhPDGF-BB) and pegs (160–380 μm) oriented perpendicularly to the root to simulate PDL formation. Although the case’s long-term follow-up did not show clinical success, it remains a commendable effort to clinically translate a novel tissue engineering strategy. The authors suggested improvements in the scaffold design, such as using fast resorbing materials to synchronize with the natural healing, greater surface area and more porosity to ensure higher interconnectedness of the graft with natural tissue [122]. This remains a solo effort in the clinical application of modelling and designing of 3D printed scaffold clinically to the best of our knowledge. 

There is a widespread and popular role of computer-assisted diagnostics and treatment planning, while the digital designing of scaffolds in periodontal repair is still in the preclinical developmental pipeline. The clinical usage has been translated in limited areas, namely modelling to contour grafts in required topology, auto-implantation of tooth, partial or full mouth restoration using implants. This indicates that 3D printing technology offers promising treatment options, but clinical translation requires more consorted efforts inspired by medical and other emerging bioengineering technologies.

## 9. 3D Printing in Orthodontics and Dentofacial Orthopedics

With 3D printing becoming more readily available and more research showing its accuracy, it may cause a paradigm shift in diagnosing and planning orthodontic treatment. Digitization has not only allowed us to shorten treatment time but has also provided for opportunities to customize treatments, further allowing dentists to improve diagnosis and treatment planning abilities.

Firstly, in terms of diagnosis and treatment planning in all aspects of dentistry, including orthodontics, dental models that give an accurate measurement of spacing, crowding, arch form, and dimensions are of high importance. Traditionally, these models have been poured in plaster through alginate impressions. However, with digital impressions gaining more traction, 3D printed models are becoming more useful. A study showed that digital-light processing is a technique that allows for printing accurate 3D models and has shown high reproducibility [123]. In another study, it was shown that digital printing using digital light processing and polyjet printing techniques produced clinically adequate models with high accuracy [124]. Digital services are also easily accessible through smart phones. In a study by Morris et al. (2019) it was shown that the Dental Monitoring smartphone application could be used to create 3D digital dental models by integrating photographs and videos, and these models are accurate enough to be used for clinical applications [125].

Using virtual surgical planning and 3D templates for orthognathic surgeries has gained more popularity due to their ease of use, feasibility, and predictable outcomes [126]. Using 3D CT imaging can help diagnose facial asymmetry and help surgeons evaluate the severity of the asymmetry as well [127]. This can help construct 3D printed surgical templates to carry out the appropriate treatment to guide the osteotomy line and increase the accuracy and predictability [128,129]. 

However, there are limitations to using 3D printing and 3D models, and therefore, they should be used with care. A study showed that printed digital models with stereolithography with a horseshoe-shaped base could not replace conventional models due to their transversal contraction [130]. Another study showed that teeth 3D setup printed with fused deposition modelling are not comparable to conventional setup techniques [131]. Hassan et al. (2017) showed that models constructed using Z printer 450 were not comparable with conventional models in terms of measuring crowding, regardless of the degree of crowding [132]. 3D printing has also allowed orthodontic treatment to shift in terms of orthodontics devices. For example, clear aligners for orthodontic correction use a series of computer-generated custom plastic aligners to gradually guide the teeth into proper alignment [133]. Clear aligners have been used for mandibular prognathism correction in both surgery-first and after approaches [133]. Literature evidence show that resin-based clear dental aligners not only were more geometrically accurate but also had better mechanical properties as compared to the conventional thermoplastic-based aligners [134].

Moreover, in the future, we may be able to offer better treatment through customized appliances and individualized orthodontic treatment. Detailed scans and records can be taken and uploaded into a software which can then simulate treatment virtually [135]. Subsequently, individualized appliances can be constructed, including aligners, brackets, and archwires. In a study by Yang et al. (2019) they described a customized ceramic bracket system that were made digitally through 3D printing to give patients a unique color and shape to suit their needs, which optimized not only esthetics but also mechanics [136]. Individualized orthodontic archwires can be easily fabricated using digital models and are clinically accurate [137]. This digital technology can also be used for fabricating space maintainers in a more precise, quick and easy matter with more accuracy [138]. CAD/CAM approaches are now used to create a combined prosthetic restoration with orthodontic appliance (PROA), allowing clinicians to obtain better results with more controlled orthodontic movements [139]. These approaches also allow the fabrication of 3D metal printed mini-implant orthodontic appliances and decrease appointments [140]. They can simply transfer information to the laboratory in the form of a digital file, which reduces time and increases efficiency. However, there is still a need for more research and more progress in this area to determine their accuracy and effectiveness than standard orthodontic treatment.

3D printing has also allowed for advances in orthodontic surgery. In order to reduce orthodontic treatment time, techniques such as corticotomy accelerate orthodontics, or periodontal accelerated osteogenic orthodontics are often used [141,142]. Piezoincision is another technique that is used more recently to achieve rapid tooth movements with no trauma to periodontal support or extensive surgical trauma [143,144]. In order to make more accurate microincisions in this technique, surgical guides manufactured by the CAD/CAM technology can be used and have shown to have increased visibility, rigidity for enhanced stability and support, buccal slots for precision in location, depth and angulation, and patient comfort to aid in a minimally invasive procedure [145,146]. Using computer-based nasoalveolar moulding devices allowed for better control of the force’s magnitude and direction and minimize the time it takes to produce such devices [147]. In a study, split-type 3D printed presurgical nasoalveolar moulding was used for unilateral cleft palate patients to reduce the cleft gap and overall morphology of the nose [148]. Studies have shown that palatal plates can be digitally constructed accurately by taking digital impressions on cleft-lip babies in a non-invasive and safe manner [149]. In conclusion, 3D printing is gaining popularity due to its ease of use, time-efficiency, and accuracy. However, more research is still needed in order to determine if it can replace traditional orthodontic treatment.

## 10. 3D Printing in Dental Implantology

Tooth loss is a common problem, and the preferred treatment of choice currently is dental implantation [150]. A dental implant is placed in the jaw bone through a surgical procedure, and it is anticipated that it will anchor itself via osseointegration [151]. Osseointegration is a process through which the opposition of the bone occurs between the bone-implant interface [152]. The osseointegration process is dependent on several factors, including the patient’s age, sex, medical history, smoking behaviors, and implant dimensions [153]. The survival rate of dental implants is relatively high, although failure is also not very uncommon, occurring mostly in the elderly with negotiated bone conditions [154]. The use of metallic implants to replace lost teeth is widespread in dentistry. However, there are various issues with these implants such as the long osseointegration process, and partial osseointegration [150]. Here it should be understood that to promote osseointegration, the implant should have a porous and rough surface. This property is not present in the currently available implants in the market [155]. 

### 10.1. 3D Printing Process and Dental Implantology

The introduction of 3D printing allows the fabrication of precise and economical dental implants [156]. The 3D printing process involves designing a 3D model which is constructed by computer-aided design software. The obtained 3D model is then changed into cross-sectional parts and then directed to the 3D printer, which deposits coating after coating of the selected material to yield an item [157]. Concerning dental implants, 3D printing is usually performed to obtain a surgical guide for the future accurate placement of implants. However, scientific literature reports 3D printing to manufacture osteosynthesis plates and mandibular reconstruction as well [90]. 

### 10.2. Pros and Cons of 3D Printed Implants

The 3D printing process has several advantages, such as having a better analysis and outcome (due to easy manipulation of digital 3D models), the possibility of duplication of the process whenever needed, and cutting off on the additional operative times. The 3D printing process has certain limitations, also including the need for a skilled professional with good computing skills, immaculate planning is usually required, and an occasionally higher risk of infection for some individuals [157]. 

### 10.3. Materials Used for 3D Printing of Dental Implants

The literature reports several different materials (Table 4) used in various studies to fabricate 3D printed dental implants.

### 10.4. Evidence of the Success of 3D Printed Dental Implants in Various Settings

The scientific literature gives evidence about the success of 3D printed implants in various clinical settings. Osman et al. (2017) previously reported that 3D printed zirconia implants produced with digital processing technique (DLP) have good dimensional accuracy and mechanical properties similar to those of the conventionally produced ceramic implants [64]. Tedesco et al. (2017) compared 3D printed implants’ biocompatibility post-insertion in the tibial metaphysis of rabbits [159]. It was reported in their study that 3D printed implants demonstrated profitable bone growth and acceptable biocompatibility [159]. Chang et al. (2020) used 3D printed Bio-Active^ITRI^ dental implants (produced via laser-sintered 3D printing method) in the femur of white rabbits to analyze their performance in a large bone defect area [164]. It was reported in their study that ITRI implants demonstrated active bone formation on histomorphometric analysis [164]. Mangano et al. (2020) placed 3D-printed subperiosteal titanium implants fabricated through DMLS in the mandible of ten edentulous patients for future prosthetic restoration [160]. They reported a 100% survival rate of these implants at a one-year follow-up with minimum complications [160]. Park et al. (2020) reported a case in which a 3D printed implant was placed in a patient with an atrophic mandible due to osteoradionecrosis who received radiation treatment post squamous cell carcinoma resection [165]. Their results revealed that 3D printed implants with a pre-mounted dental implant resulted in a better mandibular function [165]. 

Considering all the advantages of 3D printed dental implants, it can be said with authority that they can lead towards better restoration, esthetics, and functionality. Their use in the past few decades has increased dramatically due to their unparalleled benefits. It is anticipated that their use would further increase in the future. They would be readily available in the clinics, and many more patients would be able to take advantage of their expedient properties.

## 11. 3D Printing in Dental Education

To prepare dental students for their first living patient, extensive hands-on training is required in a preclinical setting [166]. With computerized dentistry becoming an essential fragment of dental learning and dental practice, 3D printing has started to enhance components of dental education [167]. Regardless of progress in 3D printing, only straightforward tooth models have been available for dental students to enhance their manual skills [167]. Fortunately, recent studies have been conducted to try to satisfy the need for more natural tooth and intraoral facial models for hands-on preclinical and clinical dental training with promising results. Some of these emerging applications of 3D printing in dental education, ranging from endodontics to oral surgery training, will be discussed in this section.

Crown preparations in prosthodontics with patients are a common part of clinical preparation and standard in dental practices. Since commonly used model teeth are too uniform in color and hardness, training for a preparation technique adapted to real tooth substance is often neglected in preclinical training [168]. A study conducted by Hohne et al. (2019) from the University of Wuerzburg aimed to design and establish a 3D printed tooth with realistic differences in hardness, color, and different layers for enamel and dentin with a realistic pulp for education purposes in crown preparation [168]. To evaluate their model’s benefits, 30 experienced dentists and 38 fourth-year dental students completed a questionnaire after using their 3D printed model teeth and standard model teeth. Both the students and experts rated a positive learning experience with the 3D model tooth [168]. Hohne and Schmitter also developed another realistic tooth with carious lesions and pulp cavity through 3D printing for preclinical training [167]. In this study, the tooth was used by 47 dental students for preclinical training in caries excavation, direct capping of the pulp, crown preparation and core-build-up in 2018. Similarly, a questionnaire was given for participants. The authors concluded that the 3D-printed tooth had many features to help train dental students, and participants saw the importance of such a tooth in dental education [167]. Recent studies have also explored the possibility of 3D printed models for educational training purposes for craniofacial traumas and in dental traumatology. One study conducted by Remus et al. (2018) aimed to assess the feasibility of making a realistic 3D printed model for training in dental traumatology [166]. Their model was designed with several traumatic dental injuries based on the CBCT of the maxilla from a real patient and was reproduced using a stereolithographic printer. The authors concluded that the model was deemed relatively inexpensive and was highly useful for instructive hands-on training for undergraduate dental students [166]. Another study conducted by Nicot et al. (2019) introduced a 3D printed model to undergraduate students of an oral and maxillofacial surgery teaching program to improve the mechanical comprehension and educational aspect of craniofacial trauma [169]. The facial features were designed with data from Data Imaging and Communications in Medicine. New lesions on the Standard Tessellation Language file were created using the Computed Aided Design and Manufacturing freeware. This study concluded that their 3D printed model appears to help support craniofacial trauma education of undergraduate dental students and is relatively low-cost [169].

Furthermore, 3D printed models may also have the potential for aesthetic surgical training. In East Asia, intraoral facial skeletal contour surgeries (intraoral FSCSs) have increased in popularity to achieve an oval face. However, no intraoral FSCS simulator exists, and its existent could be beneficial for training since they are technically challenging to perform, and severe complications could arise [170]. To increase the exposure to the complicated surgery for training professionals, Fu et al. (2019) developed the first intraoral FSCS simulator and evaluated its effectiveness. The simulator was created with 3D printed skulls based on CT data of intraoral FSCS patients, and the skull was covered with elastic headcloth. The authors had 20 residents who have all previously participated in intraoral FSCSs and had them fill out questionnaires before and after practicing with the authors’ intraoral FSCS simulator. This study concluded that their intraoral FSCS simulator is effective and economical from the data collected. They also concluded that the simulator is highly realistic and has helped participants feel more confident in performing intraoral FSCSs [170].

Although 3D printed models for dental training are currently in its infancy, many models have been created using 3D technology with promising potentials. From the studies discussed, 3D models for training dental students can be used for various dental specialties and procedures while also being relatively low-cost. Compared with the uses of series models, commonly used by many universities for preclinical training, 3D printed models provide more realistic simulations [171]. However, it is essential to note that many studies on 3D printed models are new and require further testing with more participants before they may be used globally. Nevertheless, 3D printed models have proven their effectiveness in improving future and current dental professionals’ understanding and skills.

## 12. Current Challenges and Future Directions

3D printing is a technique gaining popularity in dentistry and is different from conventional subtractive computer-aided manufacturing techniques. In comparison, 3D printing is an additive process with little material waste, is more accurate, and can operate using several materials such as plastics, metals, and ceramics, which are applicable to dentistry [172]. While 3D printing has changed the workflow and has allowed for innovation in several aspects, it still faces some challenges. For example, in surgery, computed tomography and 3D printing are paving the way to produce surgical guides; however, some of the materials used may not be autoclavable and sterilizable, thus limiting their use [173]. In addition, accuracy is often dictated by the quality of the original scan taken by intraoral scanners, which remain inaccurate when taking full arch scans or surfaces with irregularities [174]. Due to the increased popularization and accessibility of 3D printing and intraoral scanners, the dental workflow is experiencing a shift to the digital realm, with many practices digitizing their patient data. The 3D printers allow dentists to print models when needed and work digitally on other cases that permit it. This shift allows a decrease need for storage space in offices. However, it increases ethical issues such as data privacy, protection, and confidentiality, especially since digitizing patient data can make it more accessible for research use [175].

## 13. Conclusions

Today, a dentist’s main challenge will be shifting to a digital workflow and integrating these new technologies and equipment into their routine practice. These tools will allow the dentist to be more creative and perform more predictable, less invasive and cost-effective treatments. However, with the addition of a new technology comes a new responsibility. New standards using the equipment will have to be defined to ensure that the patient’s standard of care, health and safety are not compromised.

## Figures and Tables

**Table 1:** Advantages and disadvantages of the most commonly used additive manufacturing (AM) methods utilized in dentistry [18,20,21,22,23].

Type of Printing Technique	Advantages	Disadvantages
SLA	- Adaptable to variable material selection - Highest resolution and accuracy - Suitable for fine details and functional prototyping	- High cost per part - Complex post processing - Biohazardous materials are used - The final part is mechanically and vertically weak - High maintenance laser
SLS	- Low cost for parts - Mechanical properties maintained for functional prototyping, - Wide range of materials	- Polymer must be in powder - Not suitable for large parts - Designs with thing walls (<1 mm) have difficulty for print - High maintenance due to potential hazard
DLP	- Simple components for the machine - One of the smoothest finishes on parts is created by DLP	- Larger parts would have lower resolution - Not suitable for surgical guides requiring high accuracy - Resolution only increases if the available build area is limited, (only visible on highly detailed models) small vertical voxel lines are created
FDM	- Low cost - No flammable material hence no risk of explosion - Suitable for complex structures - Wide range of materials	- Low accuracy and resolution - Parts would need smoothening process after the print

**Table 2:** A list of additive manufacturing (AM) technologies based on the biomaterials utilized in the field of dentistry [25,26,27,28].

AM Technology Type	Compatible Dental Materials	Approximate Accuracy *
Inkjet Printing (IJP)	Low viscosity cell slurries or polymer hydrogels	35 to 40 µm
Polyjet Printing (PJP)	Photopolymers	20 to 85 µm
Multi-Jet-Printing (MJP)	Ceramic, Metal or Plastic	25 to 35 µm
FDM	Acrylonitrile butadiene styrene (ABS), Polyesters, Polypropylene or Polycarbonate	35 to 40 µm
SLA	Ceramics, Acrylate photopolymers or Plastic	50 to 55 µm
SLS	Ceramic, Metal, Thermoplastics or Plastic	45 to 50 µm
Direct Metal Laser Sintering (DMLS) or Selective Laser Melting (SLM)	Cobalt, Titanium, Aluminum, Steel Bronze or Nickel	20 to 35 µm
Colour-Jet Printing (JCP)	Gypsum	23 to 30 µm
Electron Beam Melting (EBM)	Metal, such as titanium	40 to 50 µm
Laminated Object Manufacturing (LOM)	Metal or Plastic	60 to 70 µm

* Approximate accuracy indicates how accurate printed models reproduce the anatomy of a patient.

**Table 3:** Clinical studies using 3D printing for patient specific applications.

Surgery/Application	Prosthesis/Treatment Using 3D Printing	Ref.
Mandibular reconstruction	- Two-tiered structure device for mandibular repositioning using FFF.	[100]
	- 3D surgical modelling using open software for improving precision and reproducibility in mandibular reconstruction with FFF.	[99]
	- 3D fabrication of guides and templates for mandibular ramus and condyle reconstruction using autogenous costochondral grafts.	[101]
	- Using an in-house approach for 3D printed customized cutting guides in mandibular reconstruction after cancer using osteocutaneous free flaps.	[102]
	- Mandibular reconstruction using patient specific mandible reconstruction plates (PSMP) using CAD/CAM fabricated transfer keys.	[103]
	- Comparison of two types of mandible reconstructive operations with scapula and FFF procedures using 3-D models fabricated from thermoplastic materials and conventional planning surgeries.	[104]
Mandibular distraction Osteogenesis (MDO)	- 3D fabrication of precise osteotomy guides for mandibular distraction in infants with obstructive sleep apnea.	[105]
Implants	- Surgical reconstruction of mandibular defect.	[106]
	- Additively manufactured sub-periosteal jaw implant (AMSJI).	[107]
Orthognathic Surgery	- Occlusal splint fabrication	[108]
	- POSG (Personalized orthognathic surgical guide)	[109]
	- 3D printed models and templates to assist in orthognathic surgery and mandibular contour osteoplasty for treating craniofacial deformities.	[110]
Temporomandibular Joint reconstruction (TMJ)	- Fabrication of a 3D printed “Melbourne” prosthetic joint for complete TMJ replacement due to end stage osteoarthritis.	[111]
Facial Asymmetry	- Fabrication of a re-positioning instrument for genioplasty.	[112]
	- Fabrication of 3D osteotomy guide for correcting hemimandibular hyperplasia.	[113]
Auto transplantation	- Construction of 3D printed replica of donor mandibular 3rd molar tooth for replacing heavily damaged premolars.	[114]

**Table 4:** Summary of materials used in 3D-printed dental implants.

S. No.	Materials Used for 3D Printing of Dental Implants	Studies in the Literature
1	Plastic (MED690 VeroDentPlus)	Kalman, 2018 [158]
2	Stainless Steel (Duraform 316L)	Kalman, 2018 [158]
3	Zirconia	Osman et al., 2017 [64]
4	Titanium	Tedesco et al., 2017 [159]
5	Acrylic Resin	Mangano et al., 2020 [160]
6	PEEK	Xingting et al., 2019 [161]
7	Amorphous Magnesium Phosphate (AMP) blended with PEEK	Sikder et al., 2020 [162]
8	Cobalt-Chromium (Co-Cr) Alloy	Bae et al., 2020 [163]

## References

[1] Torabi K., Farjood E., Hamedani E. (2015). Rapid prototyping technologies and their applications in prosthodontics, a review of literature. J. Dent..

[2] Atala A., Forgacs G. (2019). Three-dimensional bioprinting in regenerative medicine: Reality, hype, and future. Stem Cells Transl. Med..

[3] Ventola C.L. (2014). Medical applications for 3d printing: Current and projected uses. Pharm. Ther..

[4] Haleem A., Javaid M. (2018). Role of CT and MRI in the design and development of orthopaedic model using additive manufacturing. J. Clin. Orthop. Trauma.

[5] Azari A., Nikzad S. (2009). The evolution of rapid prototyping in dentistry: A review. Rapid Prototyp. J..

[6] Mokli Y., Pfaff J., Santos D.P., Herweh C., Nagel S. (2019). Computer-aided imaging analysis in acute ischemic stroke—Background and clinical applications. Neurol. Res. Pract..

[7] Wang C., Huang W., Zhou Y., He L., He Z., Chen Z., He X., Tian S., Liao J., Lu B. (2020). 3D printing of bone tissue engineering scaffolds. Bioact. Mater..

[8] Joda T., Zarone F., Ferrari M. (2017). The complete digital workflow in fixed prosthodontics: A systematic review. BMC Oral Health.

[9] Mangano F., Shibli J.A., Fortin T. (2016). Digital dentistry: New materials and techniques. Int. J. Dent..

[10] Hoang D., Perrault D., Stevanovic M., Ghiassi A. (2016). Surgical applications of three-dimensional printing: A review of the current literature & how to get started. Ann. Transl. Med..

[11] Güth J.-F., Runkel C., Beuer F., Stimmelmayr M., Edelhoff D., Keul C. (2017). Accuracy of five intraoral scanners compared to indirect digitalization. Clin. Oral Investig..

[12] Zimmermanna M., Mehlb A. (2015). Virtual smile design systems: A current review. Int. J. Comput. Dent..

[13] Stanley M., Paz A.G., Miguel I., Coachman C. (2018). Fully digital workflow, integrating dental scan, smile design and CAD-CAM: Case report. BMC Oral Health.

[14] Etemad-Shahidi Y., Qallandar O.B., Evenden J., Alifui-Segbaya F., Ahmed K.E. (2020). Accuracy of 3-Dimensionally Printed Full-Arch Dental Models: A Systematic Review. J. Clin. Med..

[15] Shah P., Chong B.S. (2018). 3D imaging, 3D printing and 3D virtual planning in endodontics. Clin. Oral Investig..

[16] Chameettachal S., Yeleswarapu S., Sasikumar S., Shukla P., Hibare P., Bera A.S., Bojedla S.S.R., Pati F. (2019). 3D Bioprinting: Recent Trends and challenges. J. Indian Inst. Sci..

[17] Carneiro O.S., Silva A., Gomes R. (2015). Fused deposition modeling with polypropylene. Mater. Des..

[18] SLA vs. DLP: Guide to Resin 3D Printers. https://formlabs.com/blog/resin-3d-printer-comparison-sla-vs-dlp.

[19] Mu Q., Wang L., Dunn C.K., Kuang X., Duan F., Zhang Z., Qi H.J., Wang T. (2017). Digital light processing 3D printing of conductive complex structures. Addit. Manuf..

[20] Ackerman S., Aguilera F.C., Buie J.M., Glickman G.N., Umorin M., Wang Q., Jalali P. (2019). Accuracy of 3-dimensional-printed endodontic surgical guide: A human cadaver study. J. Endod..

[21] Buda M., Bratos M., Sorensen J.A. (2018). Accuracy of 3-dimensional computer-aided manufactured single-tooth implant definitive casts. J. Prosthet. Dent..

[22] Zhang Z.C., Li P.L., Chu F.T., Shen G. (2019). Influence of the three-dimensional printing technique and printing layer thickness on model accuracy. J. Orofac. Orthop..

[23] Yang W.F., Choi W.S., Leung Y.Y., Curtin J.P., Du R., Zhang C.Y., Chen X.S., Su Y.X. (2018). Three-dimensional printing of patient-specific surgical plates in head and neck reconstruction: A prospective pilot study. Oral Oncol..

[24] Javaid M., Haleem A. (2019). Current status and applications of additive manufacturing in dentistry: A literature-based review. J. Oral Biol. Craniofac. Res..

[25] Malik H.H., Darwood A.R.J., Shaunak S., Kulatilake P., El-Hilly A.A., Mulki O., Baskaradas A. (2015). Three-dimensional printing in surgery: A review of current surgical applications. J. Surg. Res..

[26] Barazanchi A., Li K.C., Al-Amleh B., Lyons K., Waddell J.N. (2017). Additive technology: Update on current materials and applications in dentistry. J. Prosthodont..

[27] Vasamsetty P., Pss T., Kukkala D., Singamshetty M., Gajula S. (2020). 3D printing in dentistry—Exploring the new horizons. Mater. Today.

[28] Obregon F., Vaquette C., Ivanovski S., Hutmacher D.W., Bertassoni L.E. (2015). Three-dimensional bioprinting for regenerative dentistry and craniofacial tissue engineering. J. Dent. Res..

[29] Annabi N., Tamayol A., Uquillas J.A., Akbari M., Bertassoni L.E., Cha C., Camci-Unal G., Dokmeci R., Peppas N.A., Khademhossaini A. (2014). 25th anniversary article: Rational design and applications of hydrogels in regenerative medicine. Adv. Mater..

[30] Bajaj P., Schweller R.M., Khademhosseini A., West J.L., Bashir R. (2014). 3D biofabrication strategies for tissue engineering and regenerative medicine. Annu. Rev. Biomed. Eng..

[31] Mantha S., Pillai S., Khayambashi P., Upadhyay A., Zhang Y., Tao O., Pham H.M., Tran S.D. (2019). Smart hydrogels in tissue engineering and regenerative medicine. Materials.

[32] Derby B. (2012). Printing and prototyping of tissues and scaffolds. Science.

[33] Stansbury J.W., Idacavage M.J. (2016). 3D printing with polymers: Challenges among expanding options and opportunities. Dent. Mater..

[34] Van Noort R. (2012). The future of dental devices is digital. Dent. Mater..

[35] Turner B.N., Strong R., Gold S.A. (2014). A review of melt extrusion additive manufacturing processes: I. Process design and modeling. Rapid Prototyp. J..

[36] Dizon J.R.C., Espera A.H., Chen Q., Advincula R.C. (2018). Mechanical characterization of 3D-printed polymers. Addit. Manuf..

[37] Abduo J., Lyons K., Bennamoun M. (2014). Trends in computer-aided manufacturing in prosthodontics: A review of the available streams. Int. J. Dent..

[38] Bukhari S., Goodacre B.J., AlHelal A., Kattadiyil M.T., Richardson P.M. (2018). Three-dimensional printing in contemporary fixed prosthodontics: A technique article. J. Prosthet. Dent..

[39] Michna S., Wu W., Lewis J. (2005). Concentrated hydroxyapatite inks for direct-write assembly of 3-D periodic scaffolds. Biomaterials.

[40] Tarafder S., Dernell W.S., Bandyopadhyay A., Bose S. (2015). SrO- and MgO-doped microwave sintered 3D printed tricalcium phosphate scaffolds: Mechanical properties and in vivo osteogenesis in a rabbit model. J. Biomed. Mater. Res. B Appl. Biomater..

[41] Miyazaki T., Nakamura T., Matsumura H., Ban S., Kobayashi T. (2013). Current status of zirconia restoration. J. Prosthodont. Res..

[42] Khaing M.W., Fuh J.Y.H., Lu L. (2001). Direct metal laser sintering for rapid tooling: Processing and characterisation of EOS parts. J. Mater. Process. Technol..

[43] Osakada K., Shiomi M. (2006). Flexible manufacturing of metallic products by selective laser melting of powder. Int. J. Mach. Tools Manuf..

[44] Kathuria Y.P. (1999). Microstructuring by selective laser sintering of metallic powder. Surf. Coat. Technol..

[45] Frazier W. (2014). Metal additive manufacturing: A review. J. Mater. Eng. Perform..

[46] Berman B. (2012). 3-D printing: The new industrial revolution. Bus. Horiz..

[47] Padrós R., Punset Fuste M., Molmeneu M., Brizuela A., Climent M., Rupérez E., Gil F.J. (2020). Mechanical properties of cocr dental-prosthesis restorations made by three manufacturing processes. influence of the microstructure and topography. Metals.

[48] Konieczny B., Szczesio-Wlodarczyk A., Sokolowski J., Bociong K. (2020). Challenges of Co-Cr Alloy additive manufacturing methods in dentistry-the current state of knowledge (systematic review). Materials.

[49] Svanborg P., Hjalmarsson L. (2020). A systematic review on the accuracy of manufacturing techniques for cobalt chromium fixed dental prostheses. Biomater. Investig. Dent..

[50] Barazanchi A., Li K.C., Al-Amleh B., Lyons K., Waddell J.N. (2020). Adhesion of porcelain to three-dimensionally printed and soft milled cobalt chromium. J. Prosthodont. Res..

[51] Park J.M., Ahn J.S., Cha H.S., Lee J.H. (2018). Wear Resistance of 3d printing resin material opposing zirconia and metal antagonists. Materials.

[52] Zeng L., Zhang Y., Liu Z., Wei B. (2015). Effects of repeated firing on the marginal accuracy of Co-Cr copings fabricated by selective laser melting. J. Prothet. Dent..

[53] Ahn S.Y., Kim N.H., Kim S., Karabucak B., Kim E. (2018). Computer-aided Design/Computer-aided manufacturing-guided endodontic surgery: Guided osteotomy and apex localization in a mandibular molar with a thick buccal bone plate. J. Endod..

[54] Anderson J., Wealleans J., Ray J. (2018). Endodontic applications of 3D printing. Int. Endod. J..

[55] Giacomino C.M., Ray J.J., Wealleans J.A. (2018). Targeted endodontic microsurgery: A novel approach to anatomically challenging scenarios using 3-dimensional-printed guides and trephine burs-a report of 3 cases. J. Endod..

[56] Athirasala A., Tahayeri A., Thrivikraman G., Franca C.M., Monteiro N., Tran V., Ferracane J. (2018). A dentin-derived hydrogel bioink for 3D bioprinting of cell laden scaffolds for regenerative dentistry. Biofabrication.

[57] Gok T., Capar I.D., Akcay I., Keles A. (2017). Evaluation of different techniques for filling simulated c-shaped canals of 3-dimensional printed resin teeth. J. Endod..

[58] Mohmmed S.A., Vianna M.E., Penny M.R., Hilton S.T., Mordan N.J., Knowles J.C. (2018). Investigations into in situ enterococcus faecalis biofilm removal by passive and active sodium hypochlorite irrigation delivered into the lateral canal of a simulated root canal model. Int. Endod. J..

[59] Mai H.-N., Lee K.-B., Lee D.-H. (2017). Fit of interim crowns fabricated using photopolymer-jetting 3D printing. J. Prosthet. Dent..

[60] Alharbi N., Alharbi S., Cuijpers V., Osman R.B., Wismeijer D. (2018). Three-dimensional evaluation of marginal and internal fit of 3D-printed interim restorations fabricated on different finish line designs. J. Prosthodont. Res..

[61] Yildirim B. (2020). Effect of porcelain firing and cementation on the marginal fit of implant-supported metal-ceramic restorations fabricated by additive or subtractive manufacturing methods. J. Prosthet. Dent..

[62] Papadiochou S., Pissiotis A.L. (2018). Marginal adaptation and CAD-CAM technology: A systematic review of restorative material and fabrication techniques. J. Prosthet. Dent..

[63] Prechtel A., Stawarczyk B., Hickel R., Edelhoff D., Reymus M. (2020). Fracture load of 3D printed PEEK inlays compared with milled ones, direct resin composite fillings, and sound teeth. Clin. Oral Investig..

[64] Osman R.B., van der Veen A.J., Huiberts D., Wismeijer D., Alharbi N. (2017). 3D-printing zirconia implants; a dream or a reality? An in-vitro study evaluating the dimensional accuracy, surface topography and mechanical properties of printed zirconia implant and discs. J. Mech. Behav. Biomed. Mater..

[65] Wang W., Yu H., Liu Y., Jiang X., Gao B. (2019). Trueness analysis of zirconia crowns fabricated with 3-dimensional printing. J. Prosthet. Dent..

[66] McLaughlin J.B., Ramos V., Dickinson D.P. (2019). Comparison of fit of dentures fabricated by traditional techniques versus cad/cam technology. J. Prosthodont..

[67] Saponaro P.C., Yilmaz B., Heshmati R.H., McGlumphy E.A. (2016). Clinical performance of CAD-CAM-fabricated complete dentures: A cross-sectional study. J. Prosthet. Dent..

[68] Kalberer N., Mehl A., Schimmel M., Muller F., Srinivasan M. (2019). CAD-CAM milled versus rapidly prototyped (3D-printed) complete dentures: An in vitro evaluation of trueness. J. Prosthet. Dent..

[69] Schweiger J.S., Edelhoff D., Güth J.-F. (2018). Systematics and concepts for the digital production of complete dentures-risks and opportunities. Int. J. Comput. Dent..

[70] Schweiger J., Guth J.F., Edelhoff D., Stumbaum J. (2017). Virtual evaluation for CAD-CAM-fabricated complete dentures. J. Prosthet. Dent..

[71] Lee S.J., Betensky R.A., Gianneschi G.E., Gallucci G.O. (2015). Accuracy of digital versus conventional implant impressions. Clin. Oral Implant. Res..

[72] Wilk B.L. (2015). Intraoral Digital impressioning for dental implant restorations versus traditional implant impression techniques. Compend. Contin. Educ. Dent..

[73] Clark W.A., Duqum I., Kowalski B.J. (2019). The digitally replicated denture technique: A case report. J. Esthet. Restor. Dent..

[74] Flugge T.V., Schlager S., Nelson K., Nahles S., Metzger M.C. (2013). Precision of intraoral digital dental impressions with iTero and extraoral digitization with the iTero and a model scanner. Am. J. Orthod. Dentofac. Orthop..

[75] Patzelt S.B., Vonau S., Stampf S., Att W. (2013). Assessing the feasibility and accuracy of digitizing edentulous jaws. J. Am. Dent. Assoc..

[76] Presotto A.G.C., Barao V.A.R., Bhering C.L.B., Mesquita M.F. (2019). Dimensional precision of implant-supported frameworks fabricated by 3D printing. J. Prosthet. Dent..

[77] Chung Y.J., Park J.M., Kim T.H., Ahn J.S., Cha H.S., Lee J.H. (2018). 3D printing of resin material for denture artificial teeth: Chipping and indirect tensile fracture resistance. Materials.

[78] Choi J.J.E., Uy C.E., Plaksina P., Ramani R.S., Ganjigatti R., Waddell J.N. (2020). Bond strength of denture teeth to heat-cured, cad/cam and 3d printed denture acrylics. J. Prosthodont..

[79] Saponaro P.C., Yilmaz B., Johnston W., Heshmati R.H., McGlumphy E.A. (2016). Evaluation of patient experience and satisfaction with CAD-CAM-fabricated complete dentures: A retrospective survey study. J. Prosthet. Dent..

[80] Kattadiyil M.T., Jekki R., Goodacre C.J., Baba N.Z. (2015). Comparison of treatment outcomes in digital and conventional complete removable dental prosthesis fabrications in a predoctoral setting. J. Prosthet. Dent..

[81] AlHelal A., Goodacre B.J., Kattadiyil M.T., Swamidass R. (2018). Errors associated with digital preview of computer-engineered complete dentures and guidelines for reducing them: A technique article. J. Prosthet. Dent..

[82] Abt E., Carr A.B., Worthington H.V. (2012). Interventions for replacing missing teeth: Partially absent dentition. Cochrane Database Syst. Rev..

[83] Hu F., Pei Z., Wen Y. (2019). Using Intraoral scanning technology for three-dimensional printing of kennedy class I removable partial denture metal framework: A clinical report. J. Prosthodont..

[84] Bajunaid S.O., Altwaim B., Alhassan M., Alammari R. (2019). The fit accuracy of removable partial denture metal frameworks using conventional and 3d printed techniques. J. Contemp. Dent. Pract..

[85] Chung C., Chen Y.-J., Chen P.-C., Chen C.-Y. (2015). Fabrication of PDMS passive micromixer by lost-wax casting. Int. J. Precis. Eng. Man..

[86] Negm E.E., Aboutaleb F.A., Alam-Eldein A.M. (2019). Virtual evaluation of the accuracy of fit and trueness in maxillary poly(etheretherketone) removable partial denture frameworks fabricated by direct and indirect cad/cam techniques. J. Prosthodont..

[87] Chen H., Li H., Zhao Y., Zhang X., Wang Y., Lyu P. (2019). Adaptation of removable partial denture frameworks fabricated by selective laser melting. J. Prosthet. Dent..

[88] Tasaka A., Shimizu T., Kato Y., Okano H., Ida Y., Higuchi S., Yamashita S. (2020). Accuracy of removable partial denture framework fabricated by casting with a 3D printed pattern and selective laser sintering. J. Prosthodont. Res..

[89] Wu J., Cheng Y., Gao B., Yu H. (2020). A novel digital altered cast impression technique for fabricating a removable partial denture with a distal extension. J. Am. Dent. Assoc..

[90] Louvrier A., Marty P., Barrabé A., Euvrard E., Chatelain B., Weber E., Meyer C. (2017). How useful is 3D printing in maxillofacial surgery?. J. Stomatol. Oral Maxillofac. Surg..

[91] Hidalgo D.A. (1989). Fibula free flap: A new method of mandible reconstruction. Plast. Reconstr. Surg..

[92] Fang F., Chung K.C. (2014). An evolutionary perspective on the history of flap reconstruction in the upper extremity. Hand Clin..

[93] Wei F.-C., Demirkan F., Chen H.-C., Chen I.-H. (1999). Double free flaps in reconstruction of extensive composite mandibular defects in head and neck cancer. Plast. Reconstr. Surg..

[94] Smithers F.A., Cheng K., Jayaram R., Mukherjee P., Clark J.R. (2018). Maxillofacial reconstruction using in-house virtual surgical planning. ANZ J. Surg..

[95] Momoh A.O., Yu P., Skoracki R.J., Liu S., Feng L., Hanasono M.M. (2011). A prospective cohort study of fibula free flap donor-site morbidity in 157 consecutive patients. Plast. Reconstr. Surg..

[96] Jacobs C.A., Lin A.Y. (2017). A new classification of three-dimensional printing technologies: Systematic review of three-dimensional printing for patient-specific craniomaxillofacial surgery. Plast. Reconstr. Surg..

[97] Lin A.Y., Yarholar L.M. (2020). Plastic surgery innovation with 3D printing for craniomaxillofacial operations. Mo. Med..

[98] Largo R.D., Garvey P.B. (2018). Updates in head and neck reconstruction. Plast. Reconstr. Surg..

[99] Ganry L., Quilichini J., Bandini C., Leyder P., Hersant B., Meningaud J. (2017). Three-dimensional surgical modelling with an open-source software protocol: Study of precision and reproducibility in mandibular reconstruction with the fibula free flap. Int. J. Oral Maxillofac. Surg..

[100] Kadowaki M., Kubo T., Fujikawa M., Tashima H., Nagayama H., Ishihara O., Yamada R., Otake I., Hosokawa K. (2017). A two-tiered structure device based on stereolithography for residual mandible repositioning in mandibular reconstruction with fibular flap. Microsurgery.

[101] Emodi O., Shilo D., Israel Y., Rachmiel A. (2017). Three-dimensional planning and printing of guides and templates for reconstruction of the mandibular ramus and condyle using autogenous costochondral grafts. Br. J. Oral Maxillofac. Surg..

[102] Bosc R., Hersant B., Carloni R., Niddam J., Bouhassira J., De Kermadec H., Bequignon E., Wojcik T., Julieron M., Meningaud J.-P. (2017). Mandibular reconstruction after cancer: An in-house approach to manufacturing cutting guides. Int. J. Oral Maxillofac. Surg..

[103] Mascha F., Winter K., Pietzka S., Heufelder M., Schramm A., Wilde F. (2017). Accuracy of computer-assisted mandibular reconstructions using patient-specific implants in combination with CAD/CAM fabricated transfer keys. J. Craniomaxillofac. Surg..

[104] Jacek B., Maciej P., Tomasz P., Agata B., Wiesław K., Radosław W., Filip G. (2018). 3D printed models in mandibular reconstruction with bony free flaps. J. Mater. Sci. Mater. Med..

[105] Resnick C. (2018). Precise osteotomies for mandibular distraction in infants with Robin sequence using virtual surgical planning. Int. J. Oral Maxillofac. Surg..

[106] Rachmiel A., Shilo D., Blanc O., Emodi O. (2017). Reconstruction of complex mandibular defects using integrated dental custom-made titanium implants. Br. J. Oral Maxillofac. Surg..

[107] Mommaerts M. (2017). Additively manufactured sub-periosteal jaw implants. Int. J. Oral Maxillofac. Surg..

[108] Shaheen E., Sun Y., Jacobs R., Politis C. (2017). Three-dimensional printed final occlusal splint for orthognathic surgery: Design and validation. Int. J. Oral Maxillofac. Surg..

[109] Li B., Shen S., Jiang W., Li J., Jiang T., Xia J., Shen S.G., Wang X. (2017). A new approach of splint-less orthognathic surgery using a personalized orthognathic surgical guide system: A preliminary study. Int. J. Oral Maxillofac. Surg..

[110] Xiao Y., Sun X., Wang L., Zhang Y., Chen K., Wu G. (2017). The application of 3D printing technology for simultaneous orthognathic surgery and mandibular contour osteoplasty in the treatment of craniofacial deformities. Aesthetic Plast. Surg..

[111] Ackland D.C., Robinson D., Redhead M., Lee P.V.S., Moskaljuk A., Dimitroulis G. (2017). A personalized 3D-printed prosthetic joint replacement for the human temporomandibular joint: From implant design to implantation. J. Mech. Behav. Biomed. Mater..

[112] Wang L., Tian D., Sun X., Xiao Y., Chen L., Wu G. (2017). The precise repositioning instrument for Genioplasty and a three-dimensional printing technique for treatment of complex facial asymmetry. Aesthetic Plast. Surg..

[113] Hatamleh M.M., Yeung E., Osher J., Huppa C. (2017). Novel treatment planning of hemimandibular hyperplasia by the use of three-dimensional computer-aided-design and computer-aided-manufacturing technologies. J. Craniofac. Surg..

[114] Verweij J.P., Moin D.A., Wismeijer D., van Merkesteyn J.R. (2017). Replacing heavily damaged teeth by third molar autotransplantation with the use of cone-beam computed tomography and rapid prototyping. J. Oral Maxillofac. Surg..

[115] Tarsitano A., Ciocca L., Scotti R., Marchetti C. (2016). Morphological results of customized microvascular mandibular reconstruction: A comparative study. J. Craniomaxillofac. Surg..

[116] Lei L., Yu Y., Ke T., Sun W., Chen L. (2019). The application of three-dimensional printing model and platelet-rich fibrin technology in guided tissue regeneration surgery for severe bone defects. J. Oral Implantol..

[117] Tarnow D.P., Magner A.W., Fletcher P. (1992). The effect of the distance from the contact point to the crest of bone on the presence or absence of the interproximal dental papilla. J. Periodontol..

[118] Yin J., Liu D., Huang Y., Wu L., Tang X. (2017). CAD/CAM techniques help in the rebuilding of ideal marginal gingiva contours of anterior maxillary teeth: A case report. J. Am. Dent. Assoc..

[119] Wang P., Tang C., Tang Y., Wu Y. (2018). Immediate implant placement and complete mouth rehabilitation with CAD-CAM titanium frameworks and cemented crowns for a patient with severe periodontal disease: A clinical report. J. Prosthet. Dent..

[120] Oh S., Kim S., Lo H.S., Choi J.-Y., Kim H.-J., Ryu G.-J., Kim S.-Y., Choi K.-K., Kim D.-S., Jang J.-H. (2018). Virtual simulation of autotransplantation using 3-dimensional printing prototyping model and computer-assisted design program. J. Endod..

[121] Van-der-Meer W.J., Jansma J., Delli K., Livas C. (2016). Computer-aided planning and surgical guiding system fabrication in premolar autotransplantation: A 12-month follow up. Dent. Traumatol..

[122] Rasperini G., Pilipchuk S., Flanagan C., Park C., Pagni G., Hollister S., Giannobile W.V. (2015). 3D-printed bioresorbable scaffold for periodontal repair. J. Dent. Res..

[123] Sherman S.L., Kadioglu O., Currier G.F., Kierl J.P., Li J. (2020). Accuracy of digital light processing printing of 3-dimensional dental models. Am. J. Orthod. Dentofac. Orthop..

[124] Brown G.B., Currier G.F., Kadioglu O., Kierl J.P. (2018). Accuracy of 3-dimensional printed dental models reconstructed from digital intraoral impressions. Am. J. Orthod. Dentofac. Orthop..

[125] Morris R.S., Hoye L.N., Elnagar M.H., Atsawasuwan P., Galang-Boquiren M.T., Caplin J., Viana G.C., Obrez C., Kusnoto C. (2019). Accuracy of dental monitoring 3D digital dental models using photograph and video mode. Am. J. Orthod. Dentofac. Orthop..

[126] Qin Z., Zhang Z., Li X., Wang Y., Wang P., Li J. (2019). One-Stage treatment for maxillofacial asymmetry with orthognathic and contouring surgery using virtual surgical planning and 3D-printed surgical templates. J. Plast. Reconstr. Aesthet. Surg..

[127] Hwang H.S., Hwang C.H., Lee K.H., Kang B.C. (2006). Maxillofacial 3-dimensional image analysis for the diagnosis of facial asymmetry. Am. J. Orthod. Dentofac. Orthop..

[128] Hara S., Mitsugi M., Kanno T., Nomachi A., Wajima T., Tatemoto Y. (2013). Three-dimensional virtual operations can facilitate complicated surgical planning for the treatment of patients with jaw deformities associated with facial asymmetry: A case report. Int. J. Oral Sci..

[129] Zinser M.J., Mischkowski R.A., Dreiseidler T., Thamm O.C., Rothamel D., Zoller J.E. (2013). Computer-assisted orthognathic surgery: Waferless maxillary positioning, versatility, and accuracy of an image-guided visualisation display. Br. J. Oral Maxillofac. Surg..

[130] Camardella L.T., Vilella O.V., van Hezel M.M., Breuning K.H. (2017). Accuracy of stereolithographically printed digital models compared to plaster models. J. Orofac. Orthop..

[131] Gonzalez Guzman J.F., Teramoto Ohara A. (2019). Evaluation of three-dimensional printed virtual setups. Am. J. Orthod. Dentofac. Orthop..

[132] Wan Hassan W.N., Yusoff Y., Mardi N.A. (2017). Comparison of reconstructed rapid prototyping models produced by 3-dimensional printing and conventional stone models with different degrees of crowding. Am. J. Orthod. Dentofac. Orthop..

[133] Kook M.S., Kim H.M., Oh H.K., Lee K.M. (2019). Clear Aligner Use Following Surgery-First Mandibular Prognathism Correction. J. Craniofac. Surg..

[134] Jindal P., Juneja M., Siena F.L., Bajaj D., Breedon P. (2019). Mechanical and geometric properties of thermoformed and 3D printed clear dental aligners. Am. J. Orthod. Dentofac. Orthop..

[135] Jheon A.H., Oberoi S., Solem R.C., Kapila S. (2017). Moving towards precision orthodontics: An evolving paradigm shift in the planning and delivery of customized orthodontic therapy. Orthod. Craniofac. Res..

[136] Yang L., Yin G., Liao X., Yin X., Ye N. (2019). A novel customized ceramic bracket for esthetic orthodontics: In vitro study. Prog. Orthod..

[137] Tavares A., Braga E., Araujo T.M. (2017). Digital models: How can dental arch form be verified chairside?. Dental Press J. Orthod..

[138] Pawar B.A. (2019). Maintenance of space by innovative three-dimensional-printed band and loop space maintainer. J. Indian Soc. Pedod. Prev. Dent..

[139] Sanchez-Monescillo A., Duarte S. (2020). PROA concept: Prosthetic restoration with orthodontic appliance. Quintessence Int..

[140] Graf S., Vasudavan S., Wilmes B. (2018). CAD-CAM design and 3-dimensional printing of mini-implant retained orthodontic appliances. Am. J. Orthod. Dentofac. Orthop..

[141] Cassetta M., Ivani M. (2017). The accuracy of computer-guided piezocision: A prospective clinical pilot study. Int. J. Oral Maxillofac. Surg..

[142] Wilcko W.M., Wilcko T., Bouquot J.E., Ferguson D.J. (2001). Rapid orthodontics with alveolar reshaping: Two case reports of decrowding. Int. J. Periodontics Restor. Dent..

[143] Dibart S., Sebaoun J.D., Surmenian J. (2009). Piezocision: A minimally invasive, periodontally accelerated orthodontic tooth movement procedure. Compend. Contin. Educ. Dent..

[144] Dibart S., Surmenian J., Sebaoun J.D., Montesani L. (2010). Rapid treatment of Class II malocclusion with piezocision: Two case reports. Int. J. Periodontics Restor. Dent..

[145] Hou H.Y., Li C.H., Chen M.C., Lin P.Y., Liu W.C., Tsai C.Y.W., Huang R.-Y. (2019). A novel 3D-printed computer-assisted piezocision guide for surgically facilitated orthodontics. Am. J. Orthod. Dentofac. Orthop..

[146] Charavet C., Lecloux G., Jackers N., Albert A., Lambert F. (2019). Piezocision-assisted orthodontic treatment using CAD/CAM customized orthodontic appliances: A randomized controlled trial in adults. Eur. J. Orthod..

[147] Hopkins B., Dean K., Appachi S., Drake A.F. (2019). Craniofacial Interventions in Children. Otolaryngol. Clin. N. Am..

[148] Zheng J., He H., Kuang W., Yuan W. (2019). Presurgical nasoalveolar molding with 3D printing for a patient with unilateral cleft lip, alveolus, and palate. Am. J. Orthod. Dentofacial Orthop..

[149] Krey K.F., Ratzmann A., Metelmann P.H., Hartmann M., Ruge S., Kordass B. (2018). Fully digital workflow for presurgical orthodontic plate in cleft lip and palate patients. Int. J. Comput. Dent..

[150] Bollman M., Malbrue R., Li C., Yao H., Guo S., Yao S. (2020). Improvement of osseointegration by recruiting stem cells to titanium implants fabricated with 3D printing. Ann. N. Y. Acad. Sci..

[151] Alghamdi H.S. (2018). Methods to improve osseointegration of dental implants in low quality (Type-IV) bone: An overview. J. Funct. Biomater..

[152] Altay M.A., Sindel A., Ozalp O., Yildirimyan N., Kader D., Bilge U., Baur D.A. (2018). Does the Intake of selective serotonin reuptake inhibitors negatively affect dental implant osseointegration? A retrospective study. J. Oral Implantol..

[153] Chrcanovic B.R., Kisch J., Albrektsson T., Wennerberg A. (2017). Analysis of risk factors for cluster behavior of dental implant failures. Clin. Implant Dent. Relat. Res..

[154] Raikar S., Talukdar P., Kumari S., Panda S.K., Oommen V.M., Prasad A. (2017). Factors affecting the survival rate of dental implants: A retrospective study. J. Int. Soc. Prev. Community Dent..

[155] Fabbro M.D., Taschieri S., Canciani E., Addis A., Musto F., Weinstein R., Dellavia C. (2017). Osseointegration of titanium implants with different rough surfaces: A histologic and histomorphometric study in an adult minipig model. Implant Dent..

[156] Dalal N., Ammoun R., Abdulmajeed A.A., Deeb G.R., Bencharit S. (2020). Intaglio surface dimension and guide tube deviations of implant surgical guides influenced by printing layer thickness and angulation setting. J. Prosthodont..

[157] Nesic D., Schaefer B.M., Sun Y., Saulacic N., Sailer I. (2020). 3D Printing approach in dentistry: The future for personalized oral soft tissue regeneration. J. Clin. Med..

[158] Kalman L. (2018). 3D printing of a novel dental implant abutment. J. Dent. Res. Dent. Clin. Dent. Prospect..

[159] Tedesco J., Lee B.E.J., Lin A.Y.W., Binkley D.M., Delaney K.H., Kwiecien J.M., Granfield K. (2017). Osseointegration of a 3D printed stemmed titanium dental implant: A pilot study. Int. J. Dent..

[160] Mangano C., Bianchi A., Mangano F.G., Dana J., Colombo M., Solop I., Admakin O. (2020). Custom-made 3D printed subperiosteal titanium implants for the prosthetic restoration of the atrophic posterior mandible of elderly patients: A case series. 3D Print. Med..

[161] Han X., Yang D., Yang C., Spintzyk S., Scheideler L., Li P., Li D., Geis-Gerstorfer J., Rupp F. (2019). Carbon fiber reinforced PEEK composites based on 3D-printing technology for orthopedic and dental applications. J. Clin. Med..

[162] Sikder P., Ferreira J.A., Fakhrabadi E.A., Kantorski K.Z., Liberatore M.W., Bottino M.C., Bhaduri S.B. (2020). Bioactive amorphous magnesium phosphate-polyetheretherketone composite filaments for 3D printing. Dent. Mater..

[163] Bae S., Hong M.-H., Lee H., Lee C.-H., Hong M., Lee J., Lee D.H. (2020). Reliability of metal 3D printing with respect to the marginal fit of fixed dental prostheses: A systematic review and meta-analysis. Materials.

[164] Chang Tu C., Tsai P.I., Chen S.Y., Kuo M.Y., Sun J.S., Chang J.Z. (2020). 3D laser-printed porous Ti6Al4V dental implants for compromised bone support. J. Formos. Med. Assoc..

[165] Park J.H., Odkhuu M., Cho S., Li J., Park B.Y., Kim J.W. (2020). 3D-printed titanium implant with pre-mounted dental implants for mandible reconstruction: A case report. Maxillofac. Plast. Reconstr. Surg..

[166] Reymus M., Fotiadou C., Hickel R., Diegritz C. (2018). 3D-printed model for hands-on training in dental traumatology. Int. Endod. J..

[167] Höhne C., Schmitter M. (2019). 3D printed teeth for the preclinical education of dental students. J. Dent. Educ..

[168] Höhne C., Schwarzbauer R., Schmitter M. (2019). 3D printed teeth with enamel and dentin layer for educating dental students in crown preparation. J. Dent. Educ..

[169] Nicot R., Druelle C., Schlund M., Roland-Billecart T., Gwénaël R., Ferri J., Gosset D. (2019). Use of 3D printed models in student education of craniofacial traumas. Dent. Traumatol..

[170] Fu X., Qiao J., Gui L., Girod S., Niu F., Liu J., Jin Q., Zhang H., Xu S., Mao X. (2019). An effective simulator for intraoral facial skeletal contour surgeries. Ann. Plast. Surg..

[171] Marty M., Broutin A., Vergnes J.N., Vaysse F. (2019). Comparison of student’s perceptions between 3D printed models versus series models in paediatric dentistry hands-on session. Eur. J. Dent. Educ..

[172] Kessler A., Hickel R., Reymus M. (2020). 3D printing in dentistry—State of the art. Oper. Dent..

[173] Dawood A., Marti Marti B., Sauret-Jackson V., Darwood A. (2015). 3D printing in dentistry. Br. Dent. J..

[174] Abduo J., Elseyoufi M. (2018). Accuracy of intraoral scanners: A systematic review of influencing factors. Eur. J. Prosthodont. Restor. Dent..

[175] Favaretto M., Shaw D., De Clercq E., Joda T., Elger B.S. (2020). Big data and digitalization in dentistry: A systematic review of the ethical issues. Int. J. Environ. Res. Public Health.

